# Pilomatricoma presenting as a giant cutaneous horn in an 8-year-old child: A case report and literature review

**DOI:** 10.1177/2050313X241284119

**Published:** 2024-09-25

**Authors:** Madeleine Crawford, Jincheng Shi, Archan Kakadekar, Ashley Sutherland

**Affiliations:** 1 Faculty of Medicine, Dalhousie University, Halifax, NS, Canada; 2 Division of Clinical Dermatology and Cutaneous Science, Department of Medicine, Dalhousie University, Halifax, NS, Canada; 3 Department of Pathology and Laboratory Medicine, Dalhousie University, Halifax, NS, Canada

**Keywords:** Perforating pilomatricoma, pilomatricomal horn, cutaneous horn, pediatric pilomatricoma

## Abstract

Pilomatricoma is an uncommon benign adnexal tumor of childhood. We report a case of pilomatricoma presenting as a large, recurrent painful cutaneous horn on the neck of an 8-year-old boy treated with surgical excision. On histopathology, classical features of pilomatricoma along with transepidermal elimination and perforation were shown. We propose that perforating pilomatricoma and pilomatrical horn represent equivalent clinical and pathological entities. The diagnosis of perforating pilomatricoma should be considered in pediatric patients presenting with a cutaneous horn.

## Introduction

Pilomatricoma, also known as pilomatrixoma, is a benign adnexal tumor originating from hair follicle matrix cells.^1^ The classic clinical manifestation is on the head and neck as a solitary, asymptomatic subcutaneous nodule without significant epidermal change.^1,2^ They are more prevalent in children and typically manifest during the first and second decades of life with over half occurring before 20 years of age, but are also observed in older adults.^2^ While the exact pathogenesis is unknown, it has been hypothesized that increased Bcl-2 expression and subsequent suppression of apoptosis in hair follicle cells result in their formation.^3^ Several clinical variants of pilomatricoma have been described, including anetoderma, proliferating, pigmented, multiple, familial, and perforating.^1^ These variants make clinical diagnosis of atypical presentations challenging, with only 16% of lesions accurately diagnosed on initial clinical exam.^1^

Perforating pilomatricoma is a rare entity that presents as crusted ulcerated nodules, or less commonly as cutaneous horns.^4^ Transepidermal elimination has been proposed as a key process in their formation. Tumor cells located in the dermis form perforating channels into the epidermal surface that correlate to the aforementioned gross appearance.^4–6^ Immunohistochemical analysis has revealed the expression of matrix metalloproteinases (MMP-9 and MMP-12) in these tumors, suggesting their role in epidermal–dermal junction degradation and superficial tumor location.^7^ Although infrequently reported, there are a small number of cases reported of this clinical variant in both children and adults.^4–6,8–10^ In this case report, we describe a pilomatricoma presenting as a cutaneous horn in an 8-year-old child with the accompanying review of the literature.

## Case report

An 8-year-old male presented to a tertiary pediatric dermatology clinic with a 6-month history of a lesion which initially began as a small firm nodule on the left lateral neck. The nodule grew and evolved over several weeks into a large keratotic nodule with an overlying cutaneous horn that was tender to touch or manipulation. On history, the lesion had episodically fallen off with associated bleeding and subsequent slow regrowth. The patient had no previous history of skin disease, and their past medical history was unremarkable.

**Figure 1.**
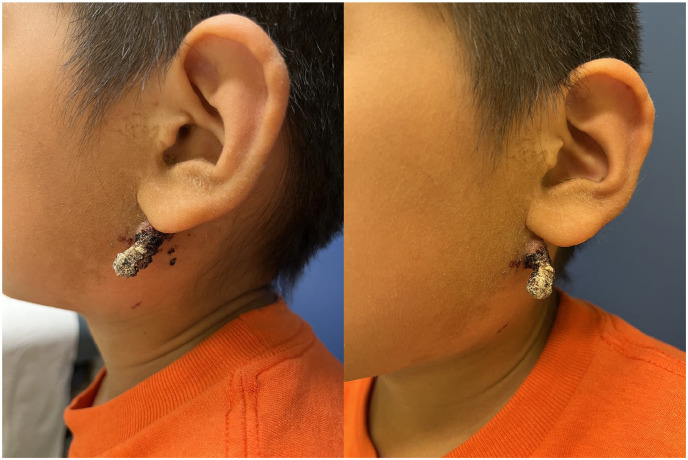
Pseudo-filiform pedunculated keratotic and crusted tumor located on the lateral neck of an 8-year-old male.

**Figure 2.**
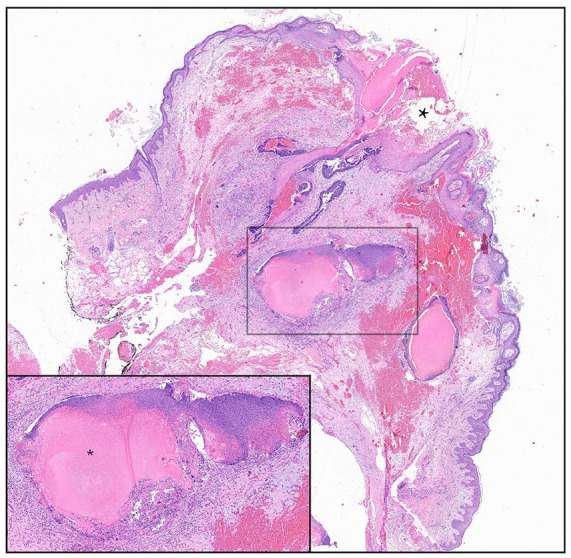
Histopathology of excised cutaneous horn showing a dermal nodule in keeping with pilomatricoma, with hair matrix cells transitioning to ghost cells (inset). Changes in transepidermal elimination corresponding to horn formation are noted (star).

Physical examination revealed a pedunculated keratotic and crusted tumor growing on the left lateral neck inferior to the earlobe (Figure 1). The lesion was approximately 2.5 cm in length, with a base 1 cm in diameter. A pyogenic granuloma or verruca vulgaris was initially suspected, and a referral was made to plastic surgery for full excision. Histopathology demonstrated a calcified dermal tumor with ghost cells, dense lymphohistiocytic infiltrate, giant cell changes, and transepidermal elimination. Clinical-pathological correlation led to a diagnosis of pilomatricoma with exuberant foreign body reaction (Figure 2).

## Discussion

The current understanding of pilomatricoma describes involvement in both children and adults but with presentation as dermal tumors rather than cutaneous horns. Cutaneous horns predominately affect the adult population between 60 and 80 years and are most commonly associated with actinic keratoses, squamous cell carcinoma, or verruca vulgaris.^11^ In the pediatric population, cutaneous horns are not commonly encountered.^12^ Possible causes in this population include irritated or giant molluscum contagiosum, verruca vulgaris, subepidermal calcified nodules, and pyogenic granulomas. Overall, there is limited recognition of pediatric cutaneous horns in literature, especially with regard to adnexal tumors including pilomatricoma.

The patient reviewed in this case was found to have a pilomatricoma manifesting as a cutaneous horn. A review of existing clinical dermatology and pathology case literature suggests that pilomatricoma may not commonly manifest as cutaneous horns since they are often located within the deep reticular dermis or subcutis tissue.^9^ The only variant of pilomatricoma known to appear as cutaneous horns is the perforating subtype, which is the least common variant in pediatric patients.^2^ Furthermore, only a few reports in the literature documented perforating pilomatricoma appearing as cutaneous horns. Between 1972 and 1986, four case reports described cutaneous horns associated with perforating pilomatricoma.^6^ Three cases involved pediatric patients under the age of 12, all of which had tumors situated in the upper portion of the dermis that extended into the epidermis through transepidermal elimination.

Perforating pilomatricomas appearing as cutaneous horns in adults are sparsely reported in existing case literature.^4,8,10^ Only one existing case report has documented a perforated cutaneous horn pilomatricoma in a pediatric patient since 1986.^5^ In 2006, de la Torre et al.^9^ proposed the term “pilomatrical horn” to describe a pilomatricoma confined to the epidermis that presented as a cutaneous horn on the arm of a 39-year-old man. They reported this as a separate entity from perforating pilomatricomas, which extend into the dermis and are associated with perforating features. We propose that “pilomatrical horn” and perforating pilomatricoma represent the same entity, with a variable anatomical location in the skin (dependent on patient age and depth of hair follicle matrix), the extent of transepidermal perforation, and reactive foreign body changes leading to hypertrophic calcification.

## Conclusion

In summary, perforating pilomatricoma appearing as a cutaneous horn is an uncommon clinical presentation that poses a diagnostic challenge due to underrepresentation in the literature. In our case, we additionally report a perforating pilomatricoma presenting as a large, recurrent, painful cutaneous horn in a pediatric patient treated successfully with surgical excision. We propose that perforating pilomatricoma and pilomatrical horn represent equivalent clinical and pathological entities and that pilomatricoma should be considered in pediatric patients presenting with a cutaneous horn.
